# Paleosecular variation recorded by Quaternary lava flows from Guadeloupe Island

**DOI:** 10.1038/s41598-018-28384-z

**Published:** 2018-07-05

**Authors:** Julia Ricci, Julie Carlut, Jean-Pierre Valet

**Affiliations:** Institut de Physique du Globe de Paris (IPGP), Sorbonne Paris Cité-Université Paris Diderot, UMR 7154 CNRS, 1 rue Jussieu, 75238, Paris, Cedex 05 France

## Abstract

Paleomagnetic directional data were obtained from fourteen 0 to 2 Ma old lava flows at Basse-Terre Island (Guadeloupe, French West Indies). Five reversed polarity flows are consistent with their Matuyama age between 1.6–1.5 Ma and 875–790 ka while the ages of the other nine normal polarity units tie them to the Olduvai subchron and the Brunhes Chron. These directions have been combined with previous results obtained from Basse-Terre Island. The overall mean direction (D = −1.2°, I = 31.4°, α_95_ = 3.3°) obtained from the 39 non-transitional flows from Basse-Terre Island is indistinguishable from the expected geocentric axial dipole value (D = 0°, I = 29.8°). The dispersion measured from the angular standard deviation of the Virtual Geomagnetic Poles (VGPs) was found to be close to, but smaller than the predictions of geomagnetic models. Together with further directions from the nearby Martinique Island, the 45 directions obtained within the Brunhes chron provide the most robust estimate of the statistical distribution of paleosecular variation (PSV) at this latitude. The sequence of directions shows episodes of high amplitude secular variation that are coeval with several geomagnetic events including the last reversal documented by five transitional directions. Finally, three lava flows have recorded a transitional behavior which could be link to two excursions, the Laguna del Sello (at ~340 ka) and the Pringle Falls (at ~210 ka) events.

## Introduction

When averaged over a long enough time interval the Earth’s magnetic field is predicted to be mostly that of a geocentric axial dipole^1,2^. However, second-order but significant deviations emerge when computing the mean direction and/or when scrutinizing the dispersion of the virtual geomagnetic poles (VGP)^3–5^. Several paleosecular variation (PSV) models have been proposed to account for these observations^6–10^, but so far none has satisfactorily accounted for all features. This may be caused by a varying fidelity of paleomagnetic records despite careful selection of the data. We must also consider bias introduced when averaging unit vectors^11^.

Geochronological studies conducted in the Martinique and Basse-Terre islands^12–20^ indicate that many lava flows belong to the Brunhes and Matuyama Chrons and therefore offer a promising potential for paleosecular variation studies. Carlut *et al*.^13^ previously studied 0–1 Ma old flows from the Basse-Terre island (Guadeloupe). These authors calculated a mean paleomagnetic pole in agreement with the geocentric axial dipole (GAD) field direction and inferred that no significant persistent second-order features were present at Basse-Terre over the last million years. Similarly, Tanty *et al*.^21^ found a mean paleomagnetic direction at Martinique island that is indistinguishable from the GAD field though the observed dispersion was higher than the PSV model predictions. In these two studies, slightly different mean inclinations were reported for the 700–400 ka and 400–0 ka periods, respectively, questioning the statistical significance of the offset^13,21^.

**Figure 1:**
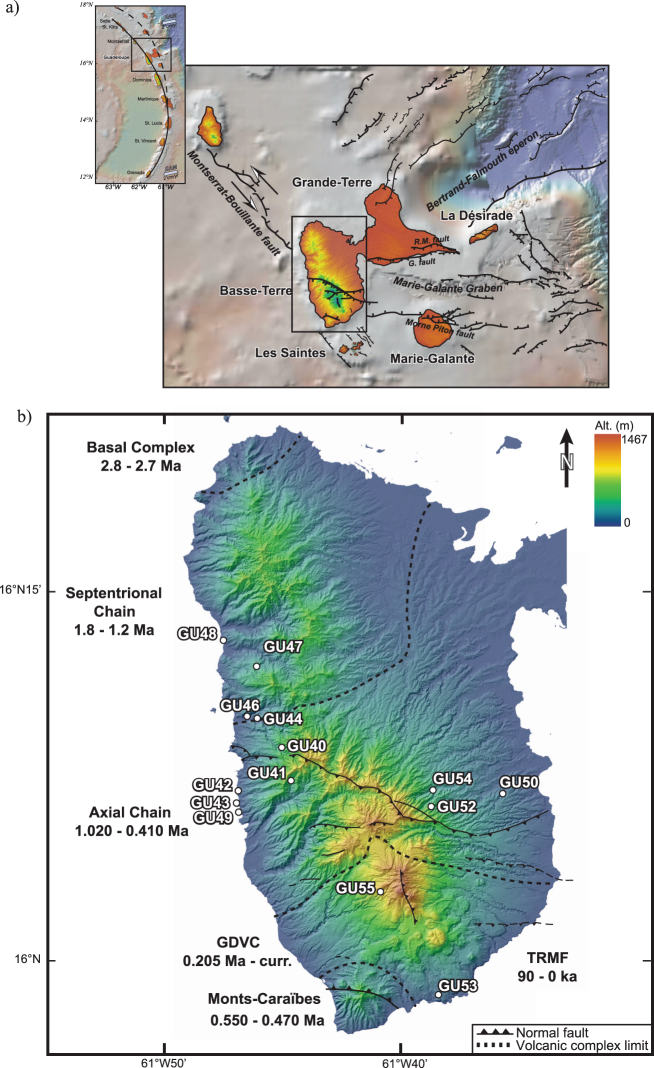
Regional Setting. (**a**) Location of Guadeloupe archipelago within Lesser Antilles arc (Black square). Continuous line: recent arc; dash line: old arc. Blue arrows: plate motion vector^65^. Regional setting of Guadeloupe archipelago with the main faults affecting the area^66^. Bathymetry image is from GeoMapApp (http://www.geomapapp.org) using bathymetry data of Smith and Sandwell^67^. Black square: location of Basse-Terre Island. (**b**) Shaded Digital Elevation Model (illumination from NW, data from the Institut Géographique National, map generated with ArcGis 10.1 software) of Basse-Terre Island, with the sites of samples analyzed in this study. K-Ar ages of the volcanic massifs compiled in Ricci *et al*.^20^.

Basse-Terre Island is located in the northern part of Lesser Antilles volcanic arc within the Guadeloupe archipelago (Fig. 1). It results from the activity of six main volcanic complexes that are characterized by an overall southward migration with lavas that are mainly basalt-andesitic to andesite^14,20^. Fourteen lava flows distributed over the whole Basse-Terre Island and with ages ranging between 1810 ka and 87 ka were sampled for paleomagnetism (Fig. 1b). At each site, between six and ten cores were drilled. All samples were magnetically oriented but sun orientation was preferably used whenever possible. We obtained new high-quality directions that improve the statistical coverage for paleosecular variation in the area. The compilation of former paleomagnetic studies^13^, added to new K-Ar radiometric ages^19,20^ provide an integrated paleomagnetic dataset for the eastern Caribbean that constrains the time-averaged field in this area.

## Results

### Paleomagnetic directions

**Figure 2:**
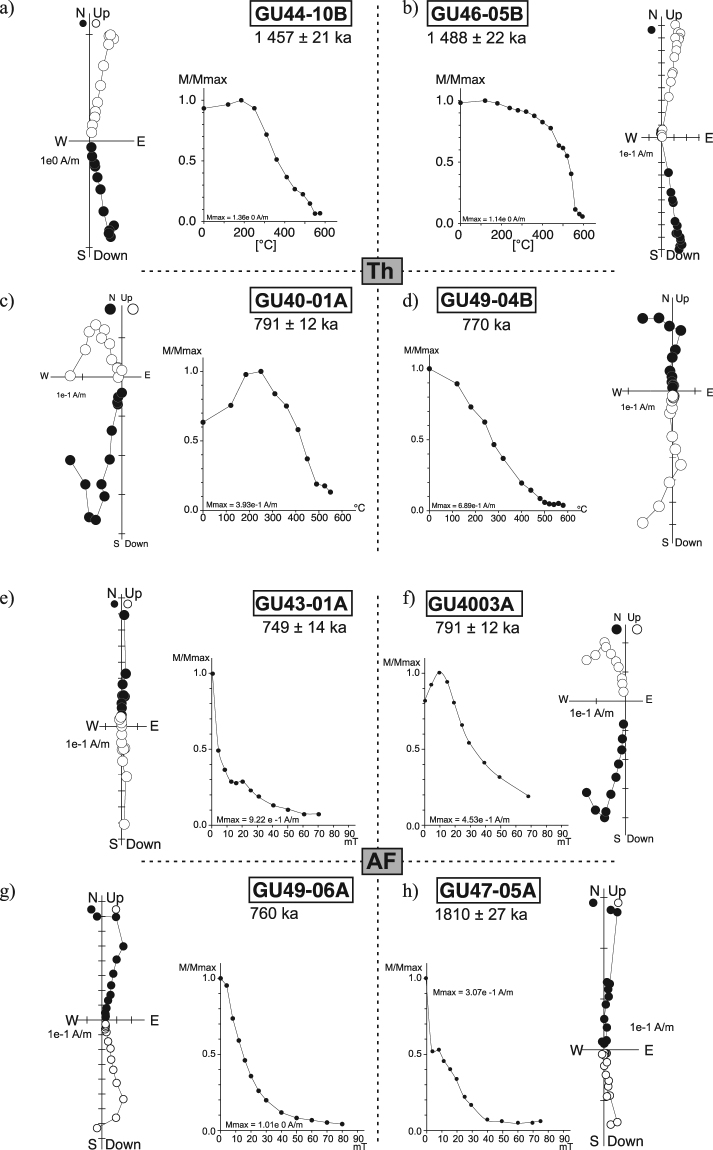
Demagnetization diagrams. Typical Zijderveld diagrams obtain by thermal (TH) (**a**–**d**) and alternating field (AF) (**e**–**h**) treatments for eight representative samples. The NRM intensity decay (M) normalized to the maximum value (Mmax) is included in the thermal and a.f. demagnetization diagrams. Solid symbols correspond to projections onto the horizontal plane, while open symbols are projections onto the vertical W-E plane.

The Characteristic Remanent Magnetization (ChRM) was determined from both alternating field (a.f.) and thermal stepwise demagnetization diagrams using principal component analysis^22,23^ and the Paleomac software^24^. Typical demagnetization diagrams for normal and reverse polarity samples are shown in Fig. 2. Most samples were totally demagnetized (i.e. with more than 95% NRM lost) after peak a.f. 70 mT or thermal demagnetization to 580 °C. In a few cases, a small residual NRM beyond 580 °C required additional temperature steps up to 610 °C. A tiny portion of Ti-hematite could be responsible for unblocking temperatures above 580 °C and stronger resistance to a.f. demagnetization. Forty per cent of samples carried a secondary magnetization component that was removed by a 15 mT a.f. or after heating at 300 °C. The characteristic direction of remanence was very well defined for ninety five percent (95%) of samples with demagnetization diagrams decreasing linearly towards the origin. The remaining 5% of the samples exhibited an erratic behavior and were therefore rejected.

**Figure 3:**
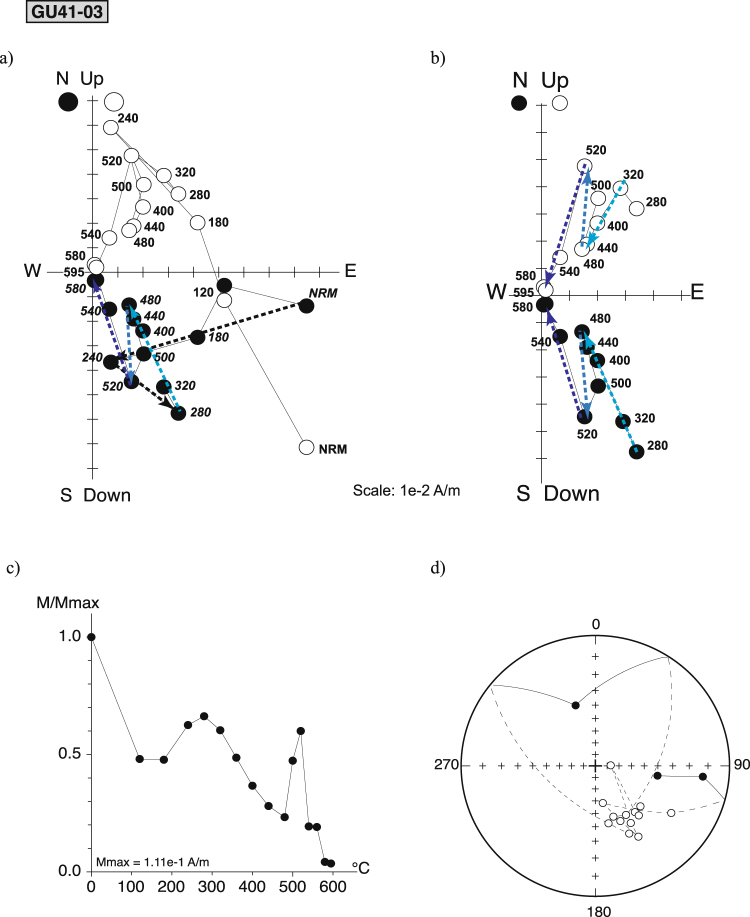
Self-reversal component (**a**) Vector component plots of directional behavior of GU41-03 highlighting a self-reversed component remove at high temperature. Arrows show the progression in thermal demagnetization. (**b**) zoom on the 280–595 °C temperature interval. (**c**) Magnetic moment decay (normalized by its maximum value) d) Stereoplot representation.

The magnetic behavior of the samples from flow GU41 showed evidence for partial self-reversal, which appeared only during thermal demagnetization (Fig. 3). In this flow, the characteristic directions of the samples were isolated beyond 500 °C and consistent with the single ChRM component obtained after a.f. demagnetization.

**Figure 4:**
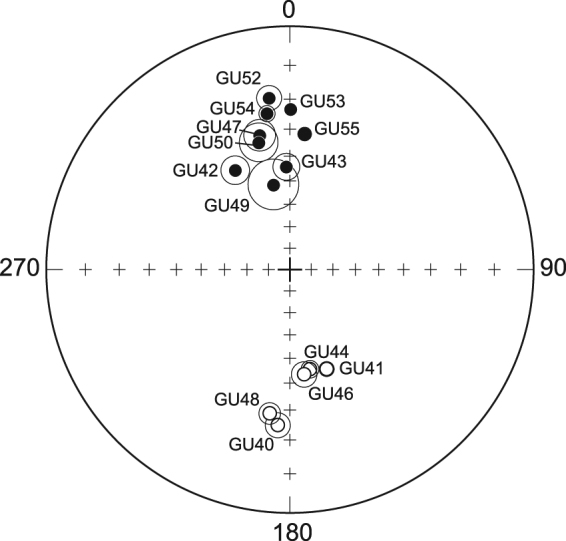
Mean paleomagnetic directions. Stereoplot (stereo projection) of the mean paleomagnetic direction obtained for each flow. Circle: α_95_, solid symbols: projections onto the horizontal plane, open symbols: projection onto the vertical plane.

The flow mean directions were obtained using Fisher statistics^25^. Data quality can be evaluated from the α_95_ confidence angle that never exceeded 8° (Table 1) and from the Fisher precision parameter k that was always greater than 63. The mean paleomagnetic directions are reported in Table 1 and plotted in Fig. 4. Five out of the fourteen lava flows have a reverse polarity (Fig. 4). The VGPs were calculated (Table 1) from the mean paleomagnetic direction of each site. The mean normal polarity VGP, located at 83.1°S, 75.5°E, is almost antipodal to the mean reverse VGP at 81.5°N, 227.7°W. All VGPs latitudes are higher than 70° (N or S), except for sites GU41 and GU42 with latitudes at 69.1° and 62.1°, respectively (Table 1).

**Table 1:** Paleomagnetic results.

Site	Locality	Longitude	Latitude	Age [ka]	n/N	Dec.	Inc	k	α95	VGP long	VGP lat.
GU47	SC	−61.76	16.21	1 810 ± 27	8/8	347.3	31.1	97.4	5.6	212.9	77.8
GU48	SC	−61.78	16.22	1671 ± 24	7/7	188.0	−28.4	261.0	3.7	215.3	−82.2
GU46	SC	−61.76	16.26	1 488 ± 22	8/8	172.3	−43.1	121.6	5.0	80.2	−78.6
GU44	SC	−61.76	16.16	1 457 ± 21	7/8	168.7	−44.7	292.6	3.5	74.0	−75.3
GU41	PB	−61.74	16.12	875 ± 21	5/8	159.8	-43.0	128.8	3.0	56.6	−69.1
GU40	PB	−61.74	16.14	791 ± 12	7/8	184.5	−24.7	211.1	4.2	244.1	−84.6
GU42	PB	−61.77	16.11	777 ± 15	5/8	331.2	40.2	192.6	5.5	226.8	62.1
GU49	PB	−61.77	16.09	770 ± 50*	5/8	349.0	51.1	88.5	8.2	267.7	71.4
GU43	PB	−61.77	16.10	759 ± 14	7/8	358.0	44.3	127.9	5.4	287.9	79.9
GU52	Mat.	−61.64	16.10	660 ± 50*	7/7	353.1	19.3	239.8	3.9	166.7	80.9
GU54	Mat.	−61.64	16.11	659 ± 11	8/8	351.6	24.2	427.6	2.7	186.3	81.2
GU50	Mat.	−61.59	16.11	616 ± 10	8/8	346.2	33.6	62.7	7.0	220.2	76.6
GU55	GDVC	−61.68	16.04	113 ± 4	11/11	6.2	31.5	356.3	2.4	17.9	84.0
GU53	GDVC	−61.64	16.97	87 ± 5	10/12	0.3	23.3	863.9	1.6	115.3	85.2

Column headings: Sample name; locality (SC: Septentrional chain; PB: Piton de Bouillante volcano; Mat.: Matéliane volcano and GDVC: Grande-Découverte volcano); Longitude and latitude coordinates; ages ±1σ uncertainty in ka, all ages from Samper *et al*.^14^ and Ricci *et al*.^20^ except GU42 dated in this study, estimated ages are indicated by a star; number of data used/total number of samples measured; Declination in degrees; Inclination in degrees; Fisher’s precision parameter; radius of the 95 per cent confidence cone from Fisher (1953); VGP longitude and latitude.

### Thermomagnetic and coercivity analyses

**Figure 5:**
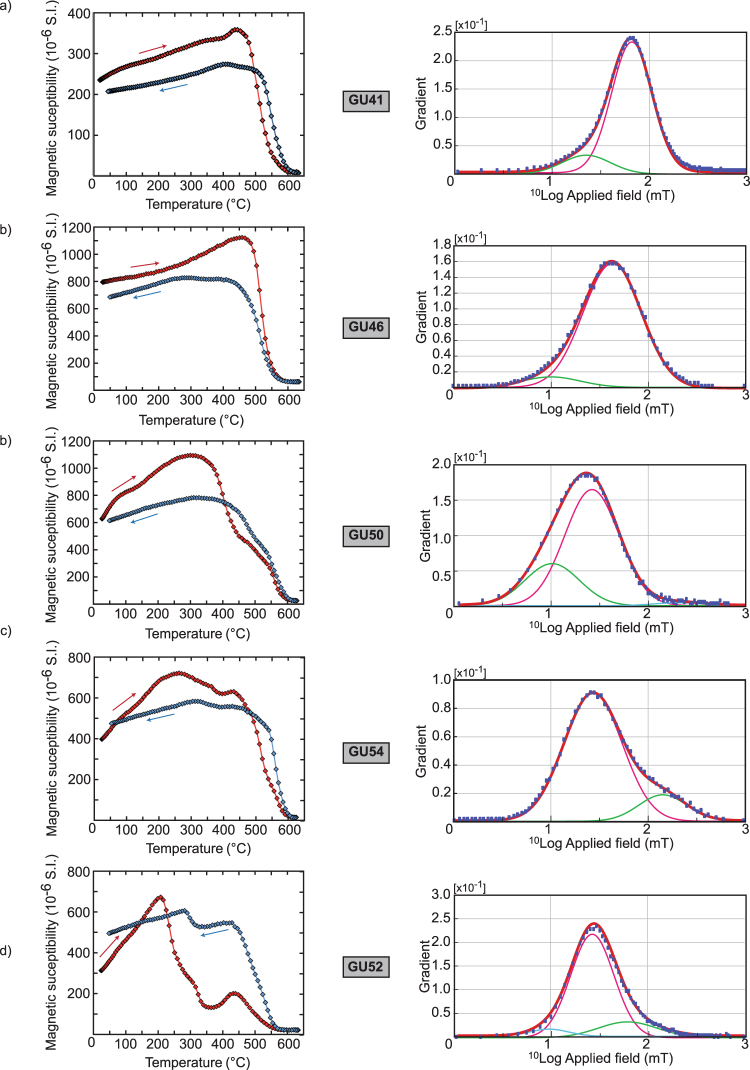
High-field rock magnetic properties. Magnetic susceptibility versus temperature (red and blue curve for heating and cooling curves, respectively), and treatment of the IRM data by the cumulative log-Gaussian (CLG) function^28^ for five representative shapes of samples studied here. Curves colored following the different magnetic components: main B_1/2_ in red, second and third in green and blue, respectively (see text for details).

Low–field susceptibility heating curves indicate that Ti–poor titanomagnetite with various degrees of Ti is the dominant carrier of remanence in all samples with the exception of GU52 (Fig. 5). A few specimens (GU40, GU42, GU44, GU46, GU47) are characterized by a single Curie temperature (Tc) between 550 and 585 °C, while others (GU41, GU43, GU48, GU49, GU50, GU53, GU54, GU55) indicate the presence of two ferromagnetic phases with a mid-Tc at about 400 °C. A few heating curves (e.g. GU49, GU54) are characterized by a large increase between 350–450 °C which was likely caused by oxidized Ti-magnetite with a high titanium content^26^. The irreversible heating and cooling curves suggest also an oxidized Ti-magnetite phase that was transformed upon heating^27^. Finally, GU52 (Fig. 5) shows an atypical strong susceptibility decrease above 200 °C that could be associated with the presence of unoxidized high Ti-Titanomagnetite. Irreversible behavior and the production of magnetite at high temperature show that some of the Ti-magnetite was likely oxidized and transformed during the experiments.

During high field acquisition experiments, most samples were saturated after exposure to a direct field of 200 mT. Unmixing of IRM curves by cumulative log-Gaussian (CLG) function^28^ isolated one to three magnetic components contributing to the high field remanence (Fig. 5 and Table 2). The main B_1/2_ component (i.e. the field at which half of the saturation isothermal remanent magnetization (SIRM) was reached; component #1 in Table 2) is associated with dispersed values of secondary B_1/2_ component ranging from ~26 to ~98 mT. A second lower coercivity component (down to ~3 mT) was also present in most samples and a higher coercivity phase with B_1/2_ values from ~126 to ~199 mT was detected in the GU54 sample and to lesser extent in GU50 and GU55. These results are interpreted in terms of mixture of titanomagnetites with varying grain-sizes, Ti-content and/or cation deficiency^28^. A fraction of titanohematite could be responsible for the higher coercivity signal in GU50, GU55 and GU54. Note that this phase was not observed during the NRM measurements and therefore has a negligible contribution to the remanence. Finally, we measured the hysteresis parameters (saturation remanence (M_rs_), saturation magnetization (M_s_), coercivity of remanence (H_cr_), and coercivity (H_c_)). The M_rs_/M_s_ versus H_cr_/H_c_ ratios are plotted in Suppl. Mat. 1. Most samples fall within the mixed Single Domain (SD) – Multi-domain (MD) curve with always less than 40% SD, except sample GU47 which displays an H_cr_/H_c_ ratio larger than 6.2 indicative of a population of MD grains^29,30^.

**Table 2:** Results of the cumulative log-Gaussian (CLG) function treatment of the IRM curve.

Site	Component	IRM contribution	(B_1/2_)	DP
		[%]	[mT]	
GU40	#1	70	93.3	0.25
	#2	30	35.5	0.34
GU41	#1	86	70.8	0.21
	#2	14	24.0	0.25
GU42	#1	96	29.5	0.37
	#2	4	3.2	0.40
GU43	#1	100	30.2	0.38
GU44	#1	68	97.7	0.23
	#2	32	41.7	0.30
GU46	#1	92	42.7	0.30
	#2	8	10.0	0.30
GU47	#1	78	43.7	0.32
	#2	22	12.6	0.40
GU48	#1	87	25.1	0.31
	#2	7	6.3	0.28
	#3	5	100.0	0.25
GU49	#1	100	36.3	0.37
GU50	#1	72	26.3	0.28
	#2	26	10.2	0.28
	#3	2	199.5	0.25
GU52	#1	78	27.5	0.22
	#2	17	63.1	0.30
	#3	5	10.0	0.20
GU53	#1	84	72.4	0.32
	#2	16	15.8	0.31
GU54	#1	86	26.9	0.30
	#2	14	141.3	0.25
GU55	#1	86	28.8	0.25
	#2	11	7.9	0.27
	#3	3	125.9	0.29

Column headings: Sample name; number of components determined; IRM contribution of each component; value of the B_1/2_ component in mT; DP value (see text for details).

### Mineralogical observations

**Figure 6:**
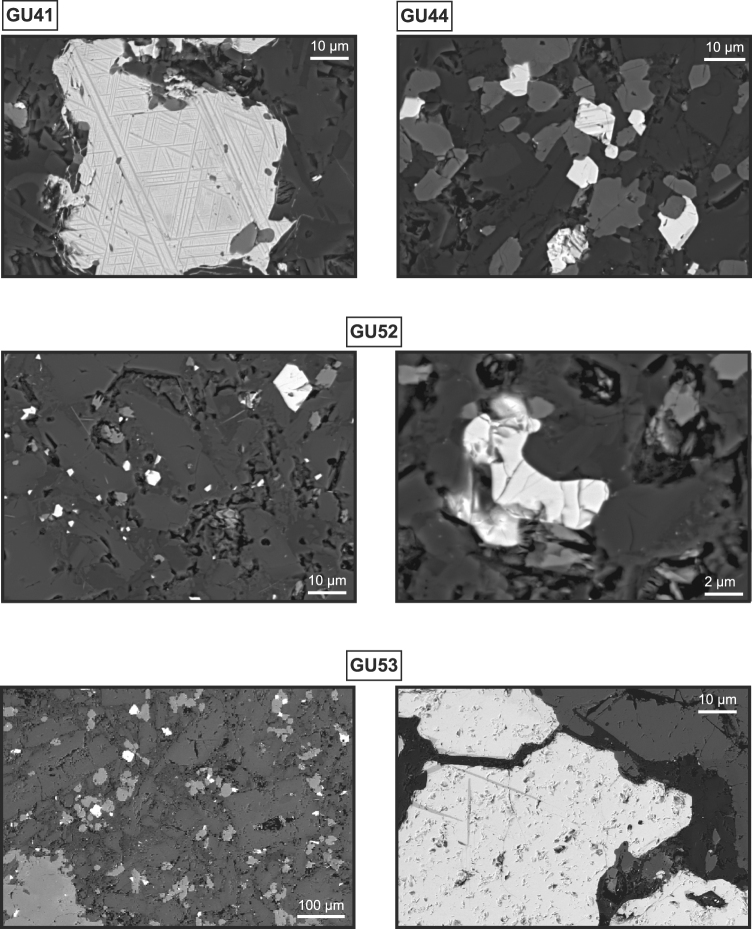
SEM images of iron oxide minerals. SEM photography of thin section for four representative samples, highlighting the exsolution of ilmenite-lamellae in titanomagnetite as well as cracks features.

The iron oxides observed with a Scanning Electronic Microscope (SEM) vary in size from sub-microns within the crystal glassy parts up to ten microns (Fig. 6). The matrix appears porous and altered for the samples GU52 and GU53, with phenocryst characterized by blunt edges. Exsolutions of ilmenite-lamellae in titanomagnetite are present in all thin sections, except for sample GU52. The EDS-X analyses of more than 50 oxides indicated a composition with various Ti-contents depending on the exsolution stage, except in sample GU52 with a rather constant composition close to Fe_2.4_Ti_0.6_O_4_ (TM60) that reveals a lack of exsolution. Multiple cracks, typical of maghemitization were also observed in this sample.

## Discussion

### Self-reversal of remanent magnetization

In rare occurrences, some rocks acquire a magnetization or partial magnetization antiparallel to the magnetic field at the time of the cooling^31–37^. Multiple antiparallel components of remanent magnetization have been identified during thermal demagnetization of the samples from the andesitic flow GU41^14^ (Fig. 3). Three opposite components were successively isolated between 280 and 595 °C from the demagnetization diagrams. The first one with reverse polarity appeared between 280 to 480 °C and was followed by a normal polarity component between 480 to 520 °C that was also characterized by an increase of the remanence. Lastly, a reverse component decreasing towards the origin of the demagnetization diagram emerged between 520 and 595 °C. Note that self-reversal process was not observed during a.f demagnetization, showing a lack of correspondence between blocking temperature and coercivity.

This 875 ± 21 ka old lava flow erupted during the Matuyama chron. Therefore, we anticipate that the reverse high temperature component recorded the ambient field direction during cooling of lava. Alteration of an initial Ti-rich titanomagnetite fraction with reverse polarity to titanomaghemite during the Brunhes chron (similarly to Hoffman’s suggestion^38^ for flows of the Liverpool volcano) could have generated a stable normal chemical remanent magnetization. However, the thermomagnetic experiments do not show any evidence for maghemite. Interestingly, some studies have highlighted self-reversal processes in andesitic and dacitic materials^35–37^ and proposed that they would be related to fine scale exsolution. However, in these cases, the antiparallel component was removed at about 300 °C, therefore at lower temperatures than in the present situation.

Alternatively, self-reversal could arise if the spontaneous magnetization of one phase reverses at a given temperature (N-type ferrimagnetism^39^) or as a two phases process by magnetostatic interaction or super-exchange coupling^34,40,41^. Such behaviors should be detected by performing continuous remanence measurements. We used the Triaxe vibrating magnetometer^42^ on small untreated and unoriented cylinders^43^ from GU41-05. We did not observe any spontaneous magnetization behavior, nor could we reproduce an antiparallel magnetization within the 480–520 °C temperature range as observed during the NRM thermal demagnetization. The very weak magnetization at this temperature (on the order of 10^−2^ Am^−1^) was at the threshold of the Triaxe sensitivity^41^ and could have thus hampered its detection. However, we rather believe that laboratory induced alteration during NRM thermal demagnetization yielded the growth of a phase with negative magnetic coupling. In contrast, this process remained negligible due to the very fast heating rate inherent to the Triaxe experiments. Several recent studies^34–37,43–45^ show that complex remanence behavior and self-reversals could be more common than previously thought in subaerial volcanic rocks, and especially in andesite, dacite and rhyolite. It is frequent however that reliable paleo-directions can be isolated at the highest temperature steps^35–37^.

### Temporal constraints and new ages

Three of the fourteen new lava flows were not dated (GU42, GU49, GU52) when the present study was initiated. We took advantage of the numerous ages now available for Basse-Terre island^20,46^ that pointed out six distinct eruptive fields with well-defined age ranges. An age estimate could thus be assigned with confidence according to the geographical location of samples. The age of ca 770 ka for GU42 and GU49 on the western flank of the Piton de Bouillante volcano was estimated from the mean age of the nearby samples distributed between 759 and 785 ka^12–14,18^. Given its rather low VGP latitude (62.1°) and proximity to the 773 ka old Matuyama-Brunhes polarity reversal (see Table 1), it was important to obtain a radiometric age for flow GU42. That also provides an opportunity to test whether the age estimate derived from the field map of Ricci *et al*.^20^ was confirmed by analytical dating. Potassium-Argon analysis was performed at the Geosciences Paris-Sud (GEOPS) Geochronology laboratory using the experimental procedure described in Ricci *et al*.^20^. The K-Ar age of 777 ± 15 ka confirmed that this flow was coeval with the last polarity reversal. Sample GU52 was collected on the eastern flank of Matéliane volcano close to GU54 (659 ± 11 ka^13^). It possibly belongs to the same volcanic episode and hence has been given an age of 660 ka. Uncertainties of ±50 ka were conservatively assigned to the ages of flows GU49 and GU52.

These new age estimates and the former ones show that the five reverse polarity flows are consistent with ages ranging from 1.6 to 1.5 Ma (for GU48, GU46 and GU44), and from 875 to 790 ka (for GU41 and GU40). Similarly, the normal polarity flow GU47 with an age of 1.810 ± 27 ka erupted during the Olduvai subchron (1.95–1.78 Ma^47^) while sites GU42, GU43, GU49, GU50, GU52, GU53, GU54 and GU55 with ages ranging from 777 to 84 ka belong to the normal polarity Brunhes period (<0.78 Ma).

### Paleosecular variations

**Figure 7:**
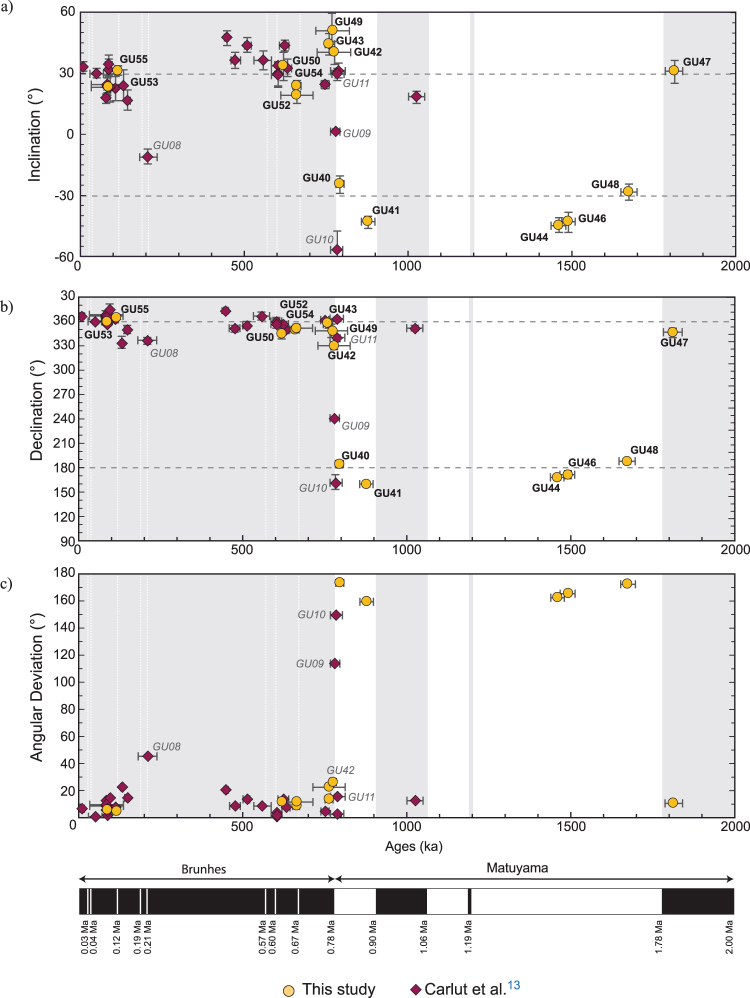
Temporal directional evolution. Temporal evolution of (**a**) inclination, (**b**) declination and (**c**) the angular deviation for Basse-Terre lava flows (Carlut *et al*.^13^; this study). The expected direction for the geocentric axial dipole field at La Guadeloupe island is shown by dashed lines. Geomagnetic polarity time scale from Gradstein *et al*.^47^.

The declination and inclination data derived from this study and Carlut *et al*.^12^ are summarized in Fig. 7. In the same figure the angular deviation from the geocentric axial dipole direction can be compared with the geomagnetic polarity timescale^47^.

Inclination data are scattered and, in some cases, strikingly different from the expected average GAD values of ±29.8° (Fig. 7a). Steeper inclinations characterize the early Brunhes (e.g. GU49, GU42) and a negative inclination is recorded at 205 ka (GU08, Fig. 7a). In order to quantify the directional scatter, found for Basse-Terre Island, we computed the angular standard deviation (ASD) and the cutoff angle which defines the boundary between directions resulting from standard paleosecular variation from transitional directions using the recursive method proposed by Vandamme^48^. The intermediate directions from flows GU09, GU10 and GU11 are related to the Matuyama-Brunhes transition^13^ and were thus excluded from the calculation. The optimum ASD is 12.1° and the corresponding cutoff angle is 26.8°. These values drop to 11.8° and 26.2°, respectively if we restrict the analysis to the Brunhes chron. The GU08 (205 ka) and GU42 (777 ka) directions with deviation angles of 30.4° and 26.3°, respectively, can both be considered as marginally transitional.

The overall mean direction D = −1.2°, I = 31.4°, α_95_ = 3.3° obtained after combining the 39 individual non-transitional directions from Basse-Terre Island is indistinguishable from the expected GAD field direction (D = 0°, I = 29.8°). The mean direction (D = −5.3°, I = 34.3°) with a larger uncertainty (α_95_ = 6.4°) obtained for the thirteen lava flows of the present study is in poorer agreement with the expected geocentric axial dipole field direction and suggests that a larger flow number is required for adequate calculation of the time-averaged field, as previously mentioned by Tanty *et al*.^21^.

**Figure 8:**
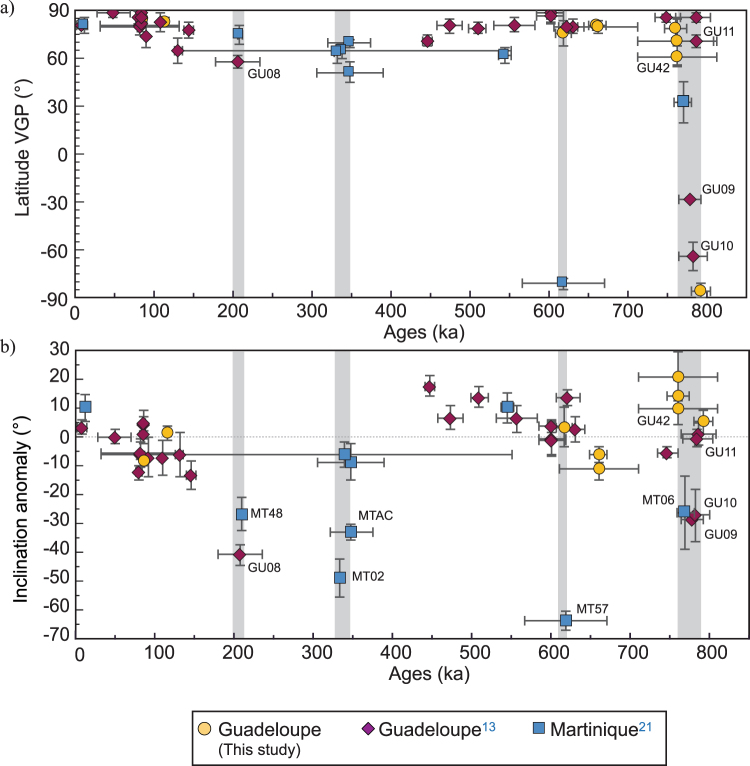
Temporal evolution of inclination and VGP. Temporal evolution during the Brunhes Chron of the (**a**) VGP latitude and (**b**) the inclination anomaly (inclination deviation from the geocentric axial dipole inclination) for samples from La Guadeloupe (Carlut *et al*.^13^, this study) and La Martinique islands^21^.

In order to integrate the Basse-Terre Island data into a more regional perspective we added the paleomagnetic directions of the individually dated flows from the nearby Martinique Island^21^. We restricted this regional dataset to the Brunhes chron (<0.78 Ma) that contains the highest measurement density with 46 independent directions. The results are presented in Fig. 8 in terms of VGP latitude and inclination anomaly (the inclination deviation from the geocentric axial dipole inclination at the site) as a function of time. Episodes of high amplitude secular variation revealed by low VGP-latitudes and/or a high inclination anomaly (Fig. 8) are indicated by shaded areas. The most striking event occurred around 770–780 ka and corresponds to the Matuyama-Brunhes polarity transition which is depicted by five independent directions (MT06 dated at 770 ± 11 ka, GU09, GU10 and GU11 with ages of 777 ± 14 ka, 781 ± 18 ka and 785 ± 22 ka, respectively, and GU42 dated at 777 ± 15 ka). The La Palma event was recorded by one flow from Martinique (MT57) and is dated at 617 ± 52 ka^21^. Two episodes with abnormal directions are found around 340 ka (331 ± 5 and 346 ± 27 ka for MT02 and MTAC, respectively) and 206 ka (GU08, 205 ± 28 ka and MT48, 207 ± 3 ka). As mentioned by Carlut *et al*.^13^ these ages could be linked to the Pringle Falls event that has been recorded at several locations worldwide and dated between 200 and 220 ka^49–51^. The Pringle Falls event has been more recently observed in sediments from site 919 in the northern Atlantic Ocean^52^. Based on anomalous inclination values, several excursions have been proposed within the 205–225 ka interval that were interpreted as a 20 kyr long period of directional instability during the low paleointensity interval documented in the East Pacific Rise sea floor magnetization^53^ as well as in the Sint-2000 and Piso-1500^54^ composite curves of relative paleointensity^55^.

In the present dataset, the 320 ka old large directional changes were only detected at Martinique. Excursion 9b discussed by Lund *et al*.^56^ has a similar age, but there is no consensus about its existence, despite one transitional flow found in Argentina (Meseta del Lago Buenos Aires) and dated by Brown *et al*.^57^ at 341 ± 33 ka (also referred as Laguna del Sello excursion by Singer^58^). We can neither completely rule out the possibility that the deviations found in Martinique were caused by displacement of large outcrops due to massive explosive events. More data are needed for better knowledge of the field instabilities during this period before inferring the existence of a geomagnetic event.

### Angular standard deviation and comparison with global models

We estimated the scatter (Sb) of VGPs caused by the variations of the Earth’s magnetic field by subtracting the within site scatter from the total measured dispersion using the formula (e.g. Johnson *et al*.^10^):

$$Sb=\sqrt{\frac{1}{N-1}\sum _{i-1}^{N}({\theta }_{i}^{2}-\frac{S{w}_{i}^{2}}{N{s}_{i}})}$$

with ${\theta }_{i}$ the angular deviation of the pole for the i^th^ site from the geographic north pole, *N* the number of sites and *Sw*_*i*_ the within-site dispersion determined from Ns_i_ samples.

**Figure 9:**
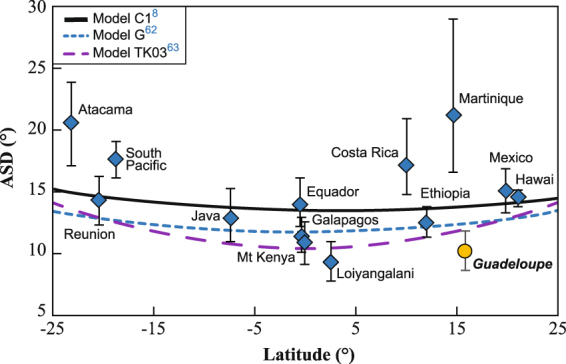
Dispersion of VGPs. Latitudinal variation (from −25 to 25°) of the angular standard deviation (ASD) of VGP for this study added to a global database^10,13,21,68–82^, and compared with proposed models (Model G^62^, Model C1^8^ and Model TK03^61^).

Considering all non-anomalous data from Basse-Terre Island, we have computed a Sb of 10.2° (Su = 12°; Sl = 8.8°, following Cox^59^) which is lower than values predicted by the G or C1 PSV models^8,60^ but closer to the trimmed scatter values (S’b) of TK03^61^ (Fig. 9). Restricting the data to the Brunhes period, we obtained a Sb of 9.6° (Su = 11.5°; Sl = 8.2°) from 32 VGPs. We believe that this Sb value for Brunhes can be seen as a reference at 16°N latitude due to the large number of independent, well dated, sites now available in Guadeloupe. Note that, it is twice lower than the dispersion of 22.3° proposed for the nearby island of La Martinique^21^. This high Sb was calculated from only 15 sites. It was likely caused by over sampling high secular variation episodes and is thus not fully representative of the time-averaged secular variation. Interestingly, the Guadeloupe Sb value is close to the low Sb of 11.2° computed from a 20°N regional compilation by Johnson *et al*.^10^ for the Brunhes period. This dispersion analysis relies on VGPs scatter in order to compare the present results with former studies, but analyzing the directions distribution could also be of interest as suggested by Linder and Gilder^62^.

## Methods

A paleomagnetic and geochronological field trip was undertaken in February 2000, in order to better understand the temporal evolution of the geomagnetic field over the last 2 Myr, as well as the volcanic evolution of Basse-Terre Island. The geochronological studies are reported in Samper *et al*.^14^. Four flows were sampled in the Septentrional Chain, five in the Piton de Bouillante volcano, and three were collected in the easternmost part of the Southern Axial Chain volcano. Lastly, two flows were sampled in the southernmost part of Basse-Terre Island (Fig. 1, Table 1). The mean local magnetic declination of −14.8° derived from the orientations of samples from 48 sites is indistinguishable from the expected International Geomagnetic Reference Field (IGRF). Preliminary paleomagnetic results of some flows, relying on only 2 to 3 samples per flow, were used by Samper *et al*.^14^ to check for consistency of geochronological data with flow polarity. However, the small number of samples as well as the absence of detailed rock magnetic investigation did not meet the requirements for a reliable paleosecular variation study.

In this study, we analyzed a total of 118 samples using both alternating fields (a.f.) and thermal stepwise demagnetization techniques in the magnetic shielded room of the Institut de Physique du Globe de Paris. Natural Remanent Magnetization was measured using Agico JR-5 and JR-6 spinner magnetometers. Eleven a.f. demagnetization steps were systematically performed at 04, 08, 12, 16, 20, 25, 30, 40, 50, 60 and 70 mT. Thermal demagnetization was carried out at 14 temperature steps (120, 180, 240, 280, 320, 360, 400, 440, 480, 500, 520, 540, 560 and 580 °C) and two additional heating steps (595 and 610 °C) were used for eleven samples.

Measurements of low field thermomagnetic susceptibility (κ) were conducted on 1 cm^3^ sample powders from each flow using an Agico KLY-3 equipped with a CS-3 furnace. Heating-cooling runs were performed in air atmosphere from 20 °C to 630 °C to detect mineralogical changes. Hysteresis loops and high-resolution acquisition curves of Isothermal Remanent Magnetization (IRM) were conducted for one representative sample from each flow using an Alternating Gradient Force Magnetometer (AGFM, Princeton Measurements Corporation) in a 1 Tesla maximum field. The IRM acquisition curves were unmixed using the CLG function^28^ in order to isolate magnetic components with different coercivities.

Finally, microscopic observation and semi-quantitative chemical data were collected on thin sections from sites GU41, GU44, GU48, GU52 and GU53 (Fig. 1b) using a scanning electronic microscope Zeiss EVO, equipped with an EDS-X detector at IPGP.

All paleomagnetic data and SEM analyses are available upon request to the authors.

## Conclusion

The paleomagnetic directional dataset for Basse-Terre Island (Guadeloupe, F.W.I) now includes 44 distinct directions from lava flows covering the last 2 Myr, of which 39 were dated by K-Ar technique. The overall mean direction (D = −1.2° and I = 31.4°) is indistinguishable from that of the geocentric axial dipole (D = 0°; I = 29.8°). The VGPs dispersion (Sb) of 10.2° and 9.6°, for the all Basse-Terre flows and only those restricted to the Brunhes chron, respectively, is lower than in PSV models (e.g. TK03^61^, C1^8^). Nevertheless, the significant number of data (N = 39 and N = 32, for all flows and Brunhes chron only, respectively), allows us to consider these values as a reference for Guadeloupe Island.

In a regional perspective, the PSV records from Guadeloupe and Martinique islands indicate an episode of high amplitude secular variation at around 205 ka that we link to the extended period of low dipole intensity during the 205–225 ka interval^49^ with one or more excursions^52^ including the Pringle Falls event^49^. This period may be representative of the “bundle” behavior suggested by Lund *et al*.^56^ characterized by a rapid succession of excursions with intervening intervals of large amplitude secular variation. The Martinique and Guadeloupe records indicate that this event was likely pronounced in the Caribbean.

Finally, we identified five flows that were erupted during the Matuyama-Brunhes transition. The weighted mean age obtained for the last reversal in the Caribbean is 777 ± 4 ka, which, considering the uncertainties, is fully compatible with the age estimate for the midpoint of the reversal at 773 ka derived from the sedimentary sequences^63,64^.

## Electronic supplementary material

Dataset 1&2
