# Supplementary material for: Paleosecular variation recorded by Quaternary lava flows from Guadeloupe Island
Source: Sci Rep. 2018 Jul 5;8:10147. doi: 10.1038/s41598-018-28384-z (PMC6033880; doi:10.1038/s41598-018-28384-z)
Supplement: Supplementary file 1 — Dataset 1&2 [file 41598_2018_28384_MOESM1_ESM.pdf]

Paleosecular variation recorded by Quaternary lava flows from Guadeloupe Island.

Julia Ricci, Julie Carlut, Jean-Pierre Valet

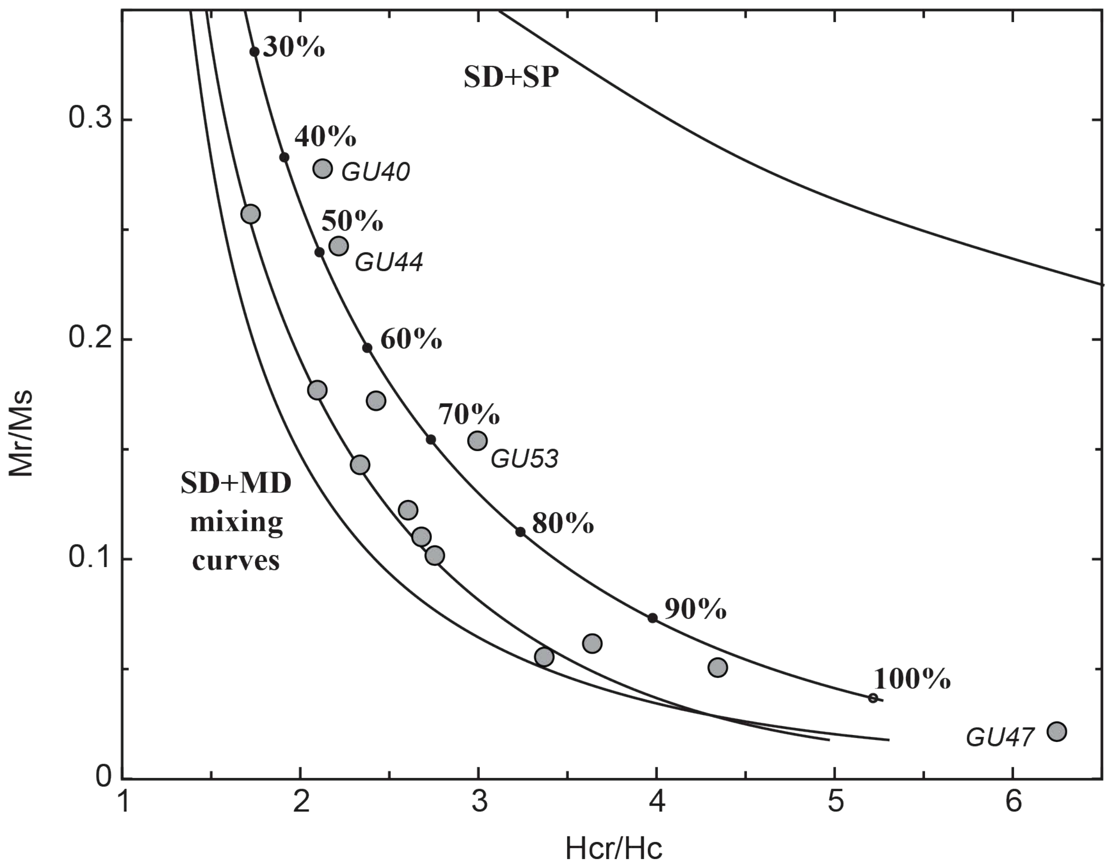

Supp. Mat. 1: Mr/Ms versus Hcr/Hc ratios for each flow[29].

Paleosecular variation recorded by Quaternary lava flows from Guadeloupe Island.

Julia Ricci, Julie Carlut, Jean-Pierre Valet

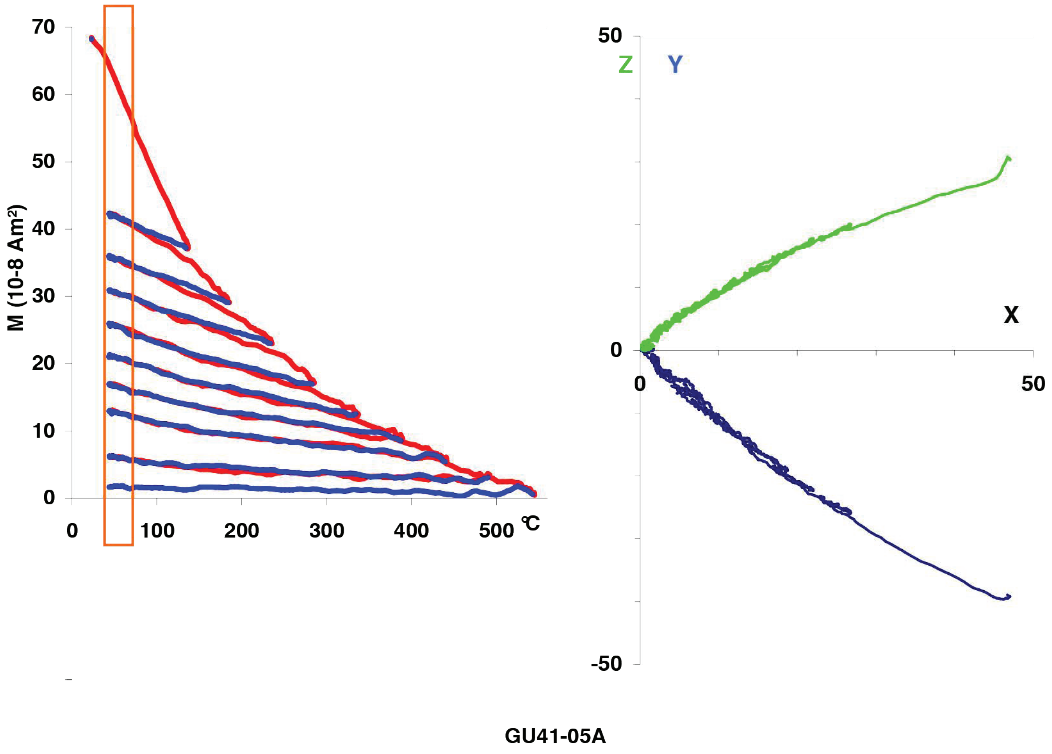

Supp. Mat. 2: Evolution of the NRM for sample GU41-05 during continuous thermal demagnetization using the Triaxe vibrating sample magnetometer44. Heating–cooling cycles are performed to progressively higher temperatures up to 550 ◦C.
